# Endothelial dysfunction: are we ready to heed the vasculature’s early-warning signal?

**Published:** 2012-05

**Authors:** Hans Strijdom

**Affiliations:** Division of Medical Physiology, Department of Biomedical Sciences, Faculty of Health Sciences, Stellenbosch University, South Africa

Endothelial dysfunction (ED) refers to a spectrum of pathophysiological changes in the vascular endothelium that ultimately results in a loss of vascular homeostasis. Traditional cardiovascular risk factors (e.g. diabetes mellitus, smoking, dyslipidaemia and hypertension) are all associated with the development of ED via sustained and harmful effects, mediated by circulating stimuli such as pro-inflammatory tumour necrosis factor-alpha (TNF-alpha), oxidised low-density lipoprotein (ox-LDL), asymmetrical dimethyl-arginine (ADMA), angiotensin II and hyperglycaemia.^1^

The underlying cellular mechanisms of ED are directly or indirectly related to the development of oxidative stress (particularly increased superoxide anion production via NADPH-oxidase and xanthine oxidase), which reduces the bioavailability of the main endothelial-derived vasodilator, nitric oxide (NO) via the reaction of superoxide with NO (thereby scavenging NO) to form peroxynitrite (ONOO^-^), a highly reactive molecule. The latter has the ability to uncouple endothelial NO synthase (eNOS), which further reduces NO production and simultaneously increases superoxide anion generation.^2^ As a result, vascular endothelial function becomes compromised, manifesting as a loss of endothelium-dependent vasorelaxation, increased thrombosis, the development of a generalised pro-inflammatory state (increased expression of vascular adhesion molecules) and increased vascular permeability.^3^ Ultimately, ED can develop into atherosclerosis.^2^

The importance of ED as a potential predictor of long-term development of atherosclerosis and cardiovascular event rate^2^ is evident by the high number of research articles on this topic (PubMed search with keywords: endothelial + dysfunction revealed 51 600 hits and approximately 3 000–4000 articles per year on this topic since 2005). Herein, however, lies both the greatest potential and challenge of current research into ED, namely translating the wealth of data obtained with laboratory-based research into scientifically validated predictive, diagnostic and even therapeutic tools in the clinical setting.

As Mudau *et al.* explain in their comprehensive review article on the cellular mechanisms and clinical applications of ED in the current issue of this journal, there is no doubt that ED serves as a crucial pathophysiological link between traditional cardiovascular risk factors and the eventual development of atherosclerosis and ischaemic heart disease (IHD).^4^ Particularly helpful in this regard is the fact that ED is an early, reversible event.^5^ This presents researchers and clinicians with a golden opportunity to not only predict the development of atherosclerosis, IHD and possibly other cardiovascular events, but also prevent the development of these conditions by therapeutically reversing early vascular dysfunction.

As Mudau and co-workers report, there are several biomarkers that could be used to assess endothelial function. However, currently elevated ADMA levels appear to be the most promising biomarker of ED in terms of prognostic value and positive correlation with cardiovascular risk. Promising data have also been obtained from studies investigating *ex-vivo* functional endothelial assessment techniques (current gold standard: flow-mediated dilatation) with regard to their ability to predict cardiovascular risk.

Despite many promising studies showing that biomarkers of ED and *ex-vivo* endothelial function assessment are strongly correlated with cardiovascular risk, a consensus for their universal clinical usefulness remains elusive.^6^ Reaching consensus is hampered by factors such as the availability of special equipment in everyday practice, the lack of data on the predictive value of more recent endothelial function assessment techniques, and the lack of studies investigating which patient groups will benefit most from *ex-vivo* endothelial function measurement.^6^ Finally, more studies demonstrating that putative anti-ED therapies actually achieve their clinical benefit by the improvement of endothelial function are required.

In the South African/African context, research into ED, its underlying mechanisms and potential clinical application is equally relevant. Indications are pointing to significant increases in the incidence of cardiovascular disease among people of African heritage in South Africa as a result of epidemiological transition and chronic diseases of lifestyle.^7^

Data from the Heart of Soweto (HOS) study in 2006 indicated a high prevalence of traditional cardiovascular risk factors such as hypertension and obesity, with almost two-thirds of the patients in the study cohort presenting with multiple risk factors.^7^ Approximately 10% of the patients in the HOS study were diagnosed with coronary artery disease. In addition, recent research has drawn attention to the interaction between HIV infection, anti-retroviral treatment and the increased rate of coronary artery disease in HIV-infected patients.^8^ Due to the fact that HIV-infected patients on treatment live longer, they are subjected to traditional cardiovascular risk factors similar to the non-infected population. However, of concern is the additional burden of non-traditional risk factors, namely the direct vascular effects of the HI virus and the anti-retroviral drugs.^7^,^8^ Consequently, HIV-infected patients on treatment now face the risk of developing premature ED and accelerated atherosclerosis.

In a recent study in South Africa, significantly higher levels of inflammatory biomarkers associated with ED were detected in newly diagnosed, untreated HIV-infected participants of African descent compared to non-infected participants, which was accompanied by age-related increases in arterial stiffness.^9^ In view of potentially unprecedented increases in ED and atherosclerosis in particularly the sub-Saharan African region, and the subsequent burden of health this could introduce, it is imperative that researchers and research funding institutions prioritise ED, with respect to both basic scientific and clinical research.

In conclusion, ED is an early and potentially reversible event in the development of atherosclerosis and can therefore be regarded as a vascular early-warning signal. As cardiovascular researchers and clinicians, we should not ignore the importance of this window of opportunity offered to us by ED, since proper detection followed by therapeutic or lifestyle interventions could prevent potentially catastrophic cardiovascular events later in the lives of affected patients.

As explained above, this has become particularly relevant in the sub-Saharan African region, as we are entering unchartered waters with regard to a predicted surge in the incidence of cardiovascular diseases previously unheard of in patients of African descent. This is due to a double burden of both traditional risk factors introduced by epidemiological transition and chronic diseases of lifestyle, but recently also due to non-traditional risk factors introduced by the HI virus and anti-retroviral drugs, particularly with regard to their targeted pro-ED effects. As basic research findings in the context of ED are moving ever closer to effective and potentially standardised clinical applications (i.e. early detection of ED followed by effective and targeted reversal of ED), it is crucial that researchers and clinicians in South Africa and Africa remain abreast of the latest developments. Are we up to the challenge?
